# The Molecular Basis of JAZ-MYC Coupling, a Protein-Protein Interface Essential for Plant Response to Stressors

**DOI:** 10.3389/fpls.2020.01139

**Published:** 2020-08-20

**Authors:** Samara Oña Chuquimarca, Sebastián Ayala-Ruano, Jonas Goossens, Laurens Pauwels, Alain Goossens, Antonio Leon-Reyes, Miguel Ángel Méndez

**Affiliations:** ^1^ Grupo de Química Computacional y Teórica, Departamento de Ingeniería Química, Universidad San Francisco de Quito USFQ, Campus Cumbayá, Quito, Ecuador; ^2^ Instituto de Simulación Computacional (ISC-USFQ), Universidad San Francisco de Quito USFQ, Quito, Ecuador; ^3^ Department of Plant Biotechnology and Bioinformatics, Ghent University, Ghent, Belgium; ^4^ VIB Center for Plant Systems Biology, Ghent, Belgium; ^5^ Laboratorio de Biotecnología Agrícola y de Alimentos, Ingeniería en Agronomía, Colegio de Ciencias e Ingenierías, Universidad San Francisco de Quito, Campus Cumbayá, Quito, Ecuador; ^6^ Colegio de Ciencias Biológicas y Ambientales COCIBA, Instituto de Microbiología, Universidad San Francisco de Quito USFQ, Campus Cumbayá, Quito, Ecuador; ^7^ Colegio de Ciencias Biológicas y Ambientales COCIBA, Instituto de Investigaciones Biológicas y Ambientales BIÓSFERA, Universidad San Francisco de Quito USFQ, Campus Cumbayá, Quito, Ecuador; ^8^ Department of Biology, The University of North Carolina at Chapel Hill, Chapel Hill, NC, United States

**Keywords:** JAZ, MYC, plant defense, machine learning, modeling, computer, hotspots

## Abstract

The jasmonic acid (JA) signaling pathway is one of the primary mechanisms that allow plants to respond to a variety of biotic and abiotic stressors. Within this pathway, the JAZ repressor proteins and the basic helix-loop-helix (bHLH) transcription factor MYC3 play a critical role. JA is a volatile organic compound with an essential role in plant immunity. The increase in the concentration of JA leads to the decoupling of the JAZ repressor proteins and the bHLH transcription factor MYC3 causing the induction of genes of interest. The primary goal of this study was to identify the molecular basis of JAZ-MYC coupling. For this purpose, we modeled and validated 12 JAZ-MYC3 3D *in silico* structures and developed a molecular dynamics/machine learning pipeline to obtain two outcomes. First, we calculated the average free binding energy of JAZ-MYC3 complexes, which was predicted to be -10.94 +/-2.67 kJ/mol. Second, we predicted which ones should be the interface residues that make the predominant contribution to the free energy of binding (molecular hotspots). The predicted protein hotspots matched a conserved linear motif SL••FL•••R, which may have a crucial role during MYC3 recognition of JAZ proteins. As a proof of concept, we tested, both *in silico* and *in vitro*, the importance of this motif on PEAPOD (PPD) proteins, which also belong to the TIFY protein family, like the JAZ proteins, but cannot bind to MYC3. By mutating these proteins to match the SL••FL•••R motif, we could force PPDs to bind the MYC3 transcription factor. Taken together, modeling protein-protein interactions and using machine learning will help to find essential motifs and molecular mechanisms in the JA pathway.

## Introduction

A considerable effort to elucidate the molecular mechanisms of plant defense has lead to a better understanding of these fascinating systems (Fürstenberg-Hägg et al., 2013; Abdul et al., 2020). The molecular mechanisms of protein-protein recognition through hormone biosynthesis and molecular signaling are not yet totally understood (Burger and Chory et al., 2019). A closer revisit could lead to valuable insights into these essential plant signaling pathways (Zhang et al., 2010; Fukao, 2012). Probably, part of the current limitations is due to the lack of structural information to study the dynamics of protein-protein interactions and the time-consuming nature experimental approaches (Xing et al., 2016). Most of the research devoted to unraveling protein interactions addresses mainly to single point mutations and deletion effects that generate invaluable information. However, these approaches can be complemented with computational biology approaches to better understand the whole dynamic process of recognition and the coupling of protein-protein complexes.

Computational biology and machine learning are excellent tools to explore the dynamics of protein-protein interactions. *In silico* experiments, using available structural and biochemical data could generate predictions of great value. In comparison with experimental approaches, they are less time-consuming, more flexible regarding size and type of proteins, and capable of providing accurate predictions of the studied system (Folador et al., 2015; Peng et al., 2017). Therefore, researchers have been using these approaches to understand complex biological problems when experimentation did not completely unravel the molecular mechanism. Examples of these studies range from protein-protein interactions for drug discovery on various diseases, including cancer (Sarvagalla et al., 2016; Goncearenco et al., 2017), neurological illnesses, metabolic disorders (Shin et al., 2017; Macalino et al., 2018), or host-pathogen protein interactions (Mariano and Wuchty, 2017), and novel interactions in plant metabolism (Ding and Kihara, 2019; Dong et al., 2019).

Under constant environmental and biotic threats, terrestrial plants have developed diverse signaling pathways to respond to the attack of arthropods, herbivores, and parasites (Turner, 2007; Koo and Howe, 2009). Tissue damage triggers plant production of small signaling molecules, namely, jasmonic acid (JA), ethylene, and salicylic acid, that lead to the modulation of transcription of genes related to the response of the plant to environmental stimuli (Pieterse et al., 2009; Wasternack and Hause, 2013).

In the JA pathway of *Arabidopsis* *thaliana*, TIFY class II Jasmonate-ZIM domain (JAZ) repressor proteins are of great importance (Pauwels and Goossens, 2011; Howe et al., 2018; Howe and Toshida, 2019). JAZ proteins can participate within a transcriptional repressor complex with the basic helix-loop-helix (bHLH) MYC transcription factor (Turner, 2007; Koo and Howe, 2009; Koo et al., 2009; Schmiesing et al., 2016). In the absence of any environmental threat, biosynthesis of the jasmonate-isoleucine (JA-Ile) hormone is low, and JAZ repressor proteins are bound to the MYC transcription factor (Chini et al., 2007; Thines et al., 2007). When the JAZ-MYC complex is formed, the co-repressors Novel Interactor of JAZ and TOPLESS bind to JAZ proteins and prevent transcription *via* histone deacetylase 6 and 19 (Chini et al., 2007; Thines et al., 2007; Yan et al., 2007; Zhang et al., 2015). Upon environmental stimuli, the levels of JA-Ile increase, and the transcriptional repressor machinery disassembles (Zhang et al., 2015). JA-Ile binds JAZ proteins and forms a complex with SCF (COI1) ubiquitin ligase, which marks JAZ proteins for degradation by the 26S proteasome (Vasyukova and Ozeretskovskaya, 2009). Meanwhile, MYC recruits mediator complex subunit 25 and the rest of the transcription machinery to begin the transcription of JA related genes such as *AOS*, *LOX2*, and *VSP2* commonly used as markers for the activation of the JA pathway (Leon-Reyes et al., 2010; Perez and Goossens, 2013; Zhang et al., 2015). It is essential to mention that all JAZ proteins have a redundant function, and that they use the same transcriptional repression mode as demonstrated the interaction with MYC (Thines et al., 2007; Demienaski et al., 2011; Withers et al., 2012; Yu et al., 2016).

MYC transcription factors, namely, MYC2, MYC3, and MYC4, regulate JA related genes involved in plant defense and plant growth. For instance, MYC2 plays a prominent role in the regulation of root growth genes such as *JAZ1* and *JAZ10* (Grunewald et al., 2009; Demianski et al., 2011). MYC3 and MYC4 are the main actors in defense of herbivory and regulation of the glucosinolate pathway (Schweizer et al., 2013). Most of the research on plant defense mechanisms has been dedicated to MYC2, with only a few studies about the molecular mechanisms of MYC3 and MYC4 (Fernández-Calvo et al., 2011; Goossens et al., 2015). Using a detailed computational approach, we investigated the molecular interactions of the TIFY class II protein family with the MYC3 transcription factor.

Machine learning approaches (also known as knowledge-based methods) for hotspot prediction is a field where outstanding contributions are currently in development, but it is still far from solved (Cukuroglu et al., 2014; Liu et al., 2018). Preceding the use of machine learning for the prediction of hotspots, the method of choice has been computational alanine scanning mutagenesis that can be implemented *via* performing full molecular dynamics simulation or using empirically calibrated free energy functions (Cukuroglu et al., 2014; Liu et al., 2018). The change in Gibbs free energy upon mutation defined the classification if the residue was a hotspot. However, threshold energy was necessary, and no universal agreement about the value existed (Liu et al., 2018). As an alternative, considering gains in speed and precision, the integration of energy terms and machine learning algorithms for hotspot predictions has been explored by several authors (Lise et al., 2009; Liu et al., 2018). In one approach, the energy terms for only the bound state of the complex were used as the input for the machine learning algorithms. The classification was based on a binary classification with a threshold of energy (ΔΔG) to dictate still the classes (Lise et al., 2009). Since the first efforts made to implement machine learning, the computational power has dramatically improved, and now the use of molecular dynamics-derived features is not as computationally expensive as once it when considered. However, it may still be challenging to apply on large-scale studies (Lise et al., 2009; Cukuroglu et al., 2014; Liu et al., 2018), and the open question remains how to effectively implement molecular dynamics with knowledge-based methods to obtain useful but still fast prediction models (Liu et al., 2018).

Here, we developed a computational workflow based on experimental data that reveals the structural basis of a transcriptional repressor complex critical in the context of plant defense response to external stimuli. Our *in silico* pipeline explored the binding thermodynamics and molecular hotspots of 14 TIFY class II protein complexes. We predicted binding energy using molecular dynamics simulations followed by free binding energy calculations. Besides, we trained a machine learning classifier to further understand each residue contribution to the binding energy in each protein-protein interface. We found significant differences in binding affinity between JAZ proteins and PPD proteins with MYC3 protein.

## Materials and Methods

### Molecular Modeling of JAZ-MYC3 Complexes

**Figure 1:**
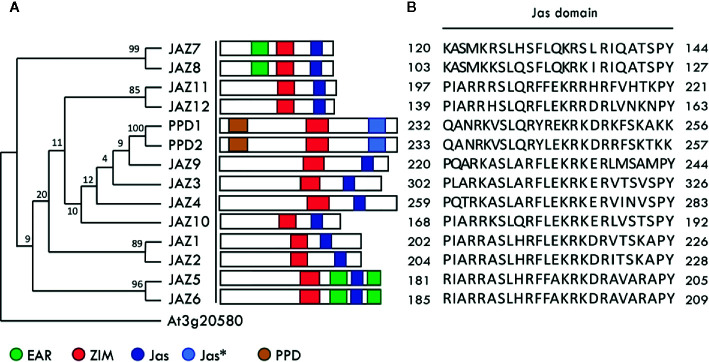
Class II TIFY protein family in Arabidopsis. **(A)** Phylogenetic tree of class II TIFY protein family in Arabidopsis. The tree was constructed using the complete amino acid sequence alignment using the maximum parsimony method (MEGA7: Molecular Evolutionary Genetics Analysis version 7.0). At3g20580 was chosen as the outgroup (Vanholme et al., 2007). The numbers above the branches are bootstrap values from 500 replicates. Graphical representations of ZIM, Jas, EAR, and PPD domains are given and were adapted from (Cuéllar Pérez et al., 2014). Jas* indicates the Jas-like domain. **(B)** Jas domain sequences from the class II TIFY protein family.

Fourteen 3D models corresponding to *Arabidopsis* JAZ-MYC3 interaction [12 models using JAZ1 to JAZ12 and MYC3] and PPD-MYC3 complexes (two models; **Figure 1**) were built using automated full-length 3D protein structural predictions using I-Tasser Service (Zhang et al., 2015). We used structural information from RCSB PDB entries 4YZ6 and 4YWC as templates to build the 3D structures. The 4YZ6 template describes the crystallographic structure of the JAZ1-MYC3 complex, which includes transcription factor MYC3 (residues 44-238) and protein JAZ1 (residues 200-221). The 4YWC template describes the JAZ9-MYC3 complex, which consists of the transcription factor MYC3 (residues 5-242) and the protein JAZ9 (residues 194-215). For further loop refinement, we use the ModLoop functionality of the Modeller v. 9.19 molecular modeling software (Eswar et al., 2003; Webb and Sali, 2014). The rationale for generating these models was to be able to adequately define the interaction network of all class II TIFY protein family members with MYC3 at an atomic level. We chose to build these models because this is the first step to study protein-protein interactions using Molecular Dynamics, which is our main computational tool in this study. Also, the 14 interfaces are not exactly the same, and each one has small differences in amino acid sequences that definitely provoke considerable differences in thermodynamic and binding properties.

### Model Quality Assessment

Before the simulation, the generated models were evaluated in terms of structural quality by ERRAT, ProQ, QMean4, and SolvX servers. Each program evaluates a different characteristic of the model, and together, they provide a general estimation of the quality of the complex (Bhutani et al., 2015). ERRAT uses an error function to statistically assess the nonbonded interactions between different atom types (Colovos and Yeates, 1993). ProQ uses neural networks to predict the structure quality regarding Solvent Accessible Surface Area (SASA), residue-residue contacts, and atom-atom contacts (Wallner and Elofsson, 2003). QMean4 checks the degree of ¨nativeness¨ of the predicted model in comparison with a data set of experimental crystal structures (Benkert et al., 2010). SolvX considers the compactness of the predicted structure by calculating the solvent accessible area, which is an indicator of proper folding (Holm and Sander, 1992).

### Molecular Dynamics Simulation (MD Simulation)

Molecular dynamics simulations of the 14 complexes were performed using Gromacs 5.1.4 with the Amber-03 force field (Berendsen et al., 1995). The system was set up as a solvated cubic box filled with SPC216 water molecules described by the TIP3P water model. Na^+^ and Cl^-^ ions were added to simulate physiological conditions at a concentration of 0.1 M. We used periodic boundary conditions and Particle-Mesh-Ewald electrostatics. The first step of the simulation was energy minimization, which was performed using the steepest descent algorithm, and a convergence parameter of less than 10 kJ mol^-1^nm^-1^. For equilibration dynamics, we ran two NVT and NPT ensemble simulations for 500 ps each. V-rescale thermostat at 310 K and Parrinello-Rahman barostat at 1 bar were used, respectively (Bhutani et al., 2015).

The equilibrated systems were subjected to a 50 ns simulation, keeping the same temperature and pressure conditions as described previously. Molecular dynamics final trajectories were analyzed using Gromacs built-in tools, namely, gmx energy, gmx rmsf, gmx rms, and gmx hbond. Clustering of the trajectory was performed using an RMSD cutoff of 0.25 nm and the gromos algorithm to determine the cluster representatives (Huber et al., 1994).

### Free Binding Energy Calculation

The free binding energy was determined using the FoldX suite functionality *AnalyseComplex.* The FoldX algorithm calculates the Gibbs free energy for each protein and then the whole complex using a linear combination of empirical terms presented in the following equations (Schymkowitz et al., 2005):

(1) $${\Delta \text{G}}_{\text{binding}}={\Delta \text{G}}_{\text{AB}}-({\Delta \text{G}}_{\text{A}}+{\Delta \text{G}}_{\text{B}})$$

ΔG_binding_ represents the change in Gibbs free energy for JAZ-MYC3 complex. Each *ΔG* term was derived from *equation 2*.

(2) $${\Delta \text{G}}_{\text{x}}=\text{a}\cdot {\Delta \text{G}}_{\text{vdw}}+\text{b}{\Delta \text{G}}_{solvH}+\text{c}\cdot {\Delta \text{G}}_{solvP}+\text{d}\cdot {\Delta \text{G}}_{\text{wd}}+\text{e}\cdot {\Delta \text{G}}_{hbOnd}+\text{f}\cdot {\Delta \text{G}}_{el}+\text{g}\cdot {\Delta \text{G}}_{\text{kon}}+\text{h}\cdot {\text{T}\Delta \text{S}}_{\text{mc}}+\text{k}\cdot {\text{T}\Delta \text{S}}_{\text{sc}}+\text{l}\cdot {\text{T}\Delta \text{G}}_{clash}$$

ΔG_x_ represents the change in Gibbs free energy for each term in *equation 1*. Each term of *equation 2* represents the contribution of Van der Waals interactions, polar, apolar solvation, hydrogen bonding, electrostatic interactions, entropic penalties, and steric overlaps present in the target system. Lower-case letters designate relative weights of each energetic term (Schymkowitz et al., 2005). We calculated the weighted binding energy average of each cluster representative for each complex. For the weighing factor, we used the number of structures in each cluster relative to the total number of structures of the stable production trajectory section.

### Machine Learning Classifier

The machine learning workflow used to predict molecular hotspots of JAZ-MYC3 was generated in Waikato Environment for Knowledge Analysis (WEKA) v.3.8.1 (Witten et al., 2016). A supervised approach was used to train the machine learning classifiers in WEKA. Multilayer Perceptron (MP), Naive Bayes (NB), Sequential minimal optimization (SMO), and Random Forest (RF) algorithms were tested for classification. The classifiers were thoroughly calibrated to assure the best predictions. The models were evaluated using model quality metrics, i.e., accuracy, recall, precision, F-measure, true-positive rate, false-positive rate, Matthew correlation coefficient, precision and recall area, and ROC area.

### Training and Validation Data Set for Machine Learning

We built a training/cross-validation data set, which comprises 19 reported cases of site-directed mutagenesis assays all belonging to the JAZ9-MYC3 complex (Melotto et al., 2008; Withers et al., 2012; Zhang et al., 2015). This data set has eleven non-deleterious mutations and eight deleterious mutations. The complete training data set is available in **Table S1** of supplementary information. FoldX suite the PSSM functionality was used to calculate energetic descriptors for the training and testing data sets (Schymkowitz et al., 2005). The PSSM tool calculates the change in free binding energy (ΔΔG) upon mutation. Computational scanning mutagenesis was performed for the 20 *natural* amino acids, and ΔΔG values were calculated.

### Experimental Validation, Gene Cloning

All cloning was carried out by Gateway^®^ recombination (Thermo Fisher Scientific, Waltham, MA, USA), as described by Goossens et al. (2015). The point mutations in PPD1(Y242F and R243L) and PPD2(Y243F) were generated with the GeneTailor^TM^ Site-Directed Mutagenesis system (Thermo Fisher Scientific) as described by Goossens et al. (2015).

### Yeast Two-Hybrid (Y2H)

Y2H analysis was performed as described in Cuéllar Pérez et al. (2014), with the GAL4 system. Briefly, bait and prey open reading frames were fused to the GAL4-AD or GAL4-BD *via* cloning into pGAL424gate or pGBT9gate, respectively. The *Saccharomyces cerevisiae* PJ69-4A yeast strain was co-transformed with bait and prey using the polyethylene glycol (PEG)/lithium acetate method. Transformants were selected on Synthetic Defined (SD) media lacking Leu and Trp (Clontech, Saint-Germain-en-Laye, France). Three individual colonies were grown overnight in liquid cultures at 30°C and 10- or 100-fold dilutions were dropped on control media (SD-Leu-Trp) and selective media lacking Leu, Trp, and His (Clontech).

## Results

### JAZ Proteins and Domains: Interactions and Pipeline

Briefly, we needed to find the structures available for the protein-protein interaction of interest and, when necessary, to generate the structure for the complexes without reported structure. Then we used these structures for the molecular dynamics simulation from which the main features for knowledge-based methods will be extracted. Finally, machine learning classifiers were trained and validated and applied to the test problem. These computational predictions were then tested in the lab and reported.

**Figure 2:**
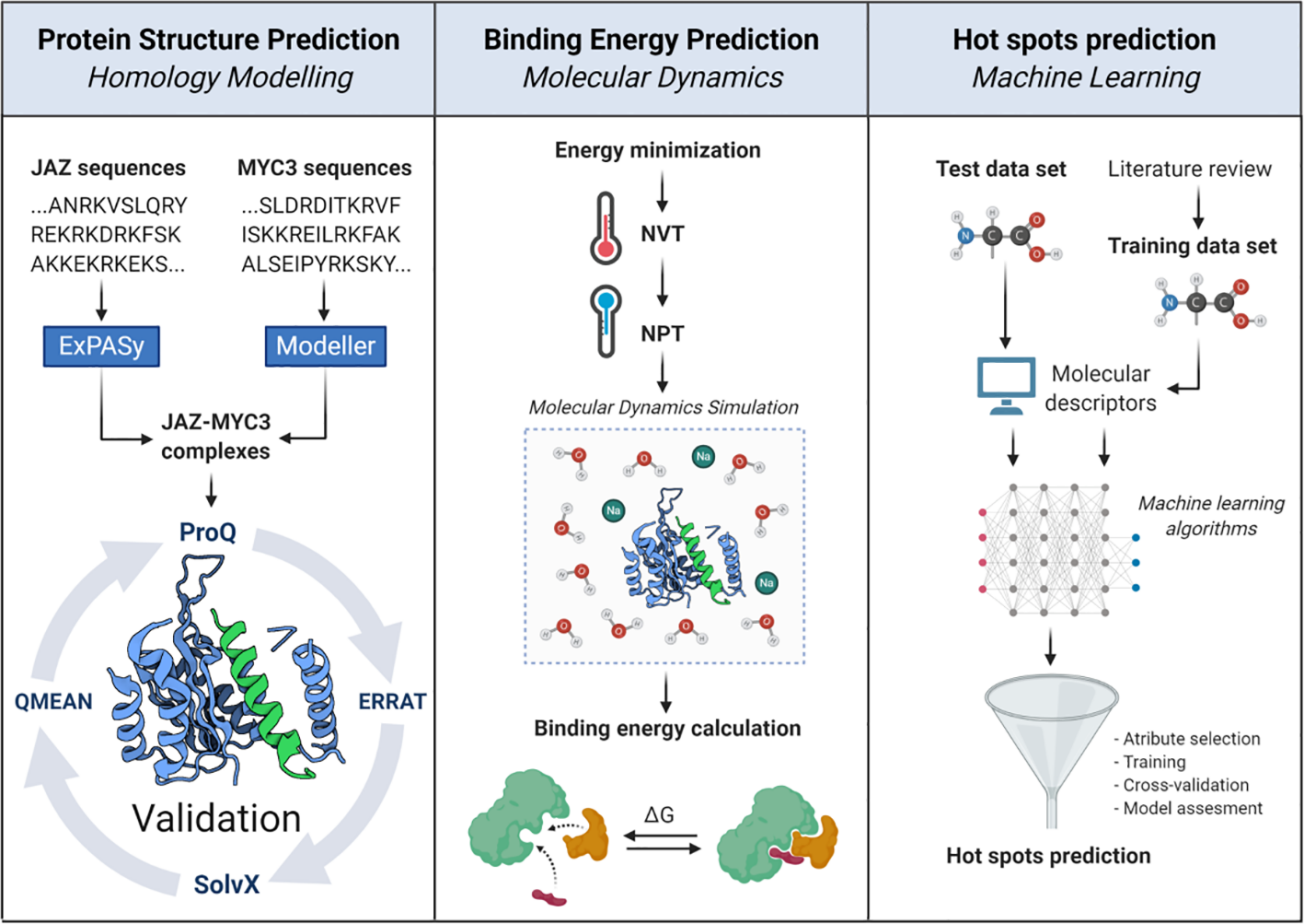
*In silico* workflow proposed to unravel structural, energetic, and molecular features of JAZ-MYC3 complexes. The pipeline is divided into three stages: protein homology modeling, binding energy calculation, and molecular hotspots discovery.

The *Arabidopsis thaliana* class II TIFY protein family comprises 12 JAZ proteins, and two non-JAZ proteins named PPD1 and PPD2 **(** **Figure 1** **).** This protein family modulates transcription by forming a transcriptional repressor complex sensitive to hormone biosynthesis. These proteins repress bHLH MYC transcription factors that regulate JA responsive genes (Fernández-Calvo et al., 2011). Yeast two-hybrid assays have proven that all JAZ proteins interact with the MYC3 transcription factor through their Jas domain (Fernández-Calvo et al., 2011; Goossens et al., 2015). The computational workflow depicted in **Figure 2** comprises three sequential stages, which allow a clear understanding of the whole interaction dynamics of JAZ-MYC3 complexes. In the first stage, we used automated full-length 3D protein structure prediction tools to generate 14 JAZ/PPD-MYC3 complexes, which were validated using different quality metrics. In the second stage, we calculated the free binding energy of each of the complexes using molecular dynamics and free energy calculations with an empirical force field. In the third stage, we detected molecular hotspots using machine learning classifiers trained with public domain site-directed mutagenesis data. The rationale behind the three stages of our computational method is multipurpose. First, a predictive model can be built if we combine molecular dynamics-derived features with experimental features from the literature. This approach does not require extensive molecular dynamics, improving the speed and the computational cost. Furthermore, by implementing experimental features and not strictly only the change in Gibbs free energy but the qualitative experimental results observed upon experimental mutations, we may capture more information than with only energy terms. Second, the data set available in general for protein-protein interfaces from experiments is still too small to generate a system capable of predicting hotspots for all cases possible in nature with the same reliability (Liu et al., 2018). Therefore, we chose the alternative of dividing the problem instead of developing a single method that predicts hotspots for all proteins. Our method will work well with a subset of similar proteins. We selected plants to test the hypothesis since the abundance of protein families with multiple closely related proteins, sometimes even almost identical copies, in the same plant. Besides, with this approach, we do not need to rely on defining a specific energy threshold to classify a residue as a hotspot or not. It may even capture relationships between neighboring residues that are hard to discern or decompose from the molecular dynamics results alone.

Finally, this hybrid computational-experimental workflow applied to class II TIFY family (JAZ-PPD proteins) will provide great insight into how the MYC3 transcription factor identifies substrates binders (JAZ) from nonsubstrates binders (PPD) at an atomic level. Using this information, we will be able to modulate the binding specificity of MYC, which will eventually lead to design of new plant immune response modulators. Finally, once we identified the key residues for the interaction, we looked to see what happened experimentally if these hotspots were inserted in PPD proteins. The wild-type PPDs lack some of the identified critical residues within their Jas-like domain. We tested PPDs mutant versions that now included the key residues, added artificially to the PPDs Jas-like domain, to see what happened with these proteins that naturally do not bind MYC3 proteins.

### JAZ-MYC3 Binding Interface

We used a server that applies a multiple threading approach that finds templates to build structural predictions. We coupled the predicted structures to a complementary stage of refinement using molecular dynamics resulting in high-quality 3D structural predictions of the 14 JAZ/PPD-MYC3 complexes. For this purpose, we performed a BLASTp query to choose our templates. BLASTp results reported five potential templates for homology modeling, as shown in **Table 1**. They corresponded to MYC3 protein-only and JAZ-MYC3 protein complexes, which have an available crystal structure in RCSB PDB. For the aim of this study, 4YWC_A and 4YZ6_A were chosen as templates since they covered the JID-TAD domain (MYC3 protein) and Jas domain (JAZ protein) which are the major interacting regions between both proteins (Zhang et al., 2015).

**Table 1:** Blastp results for MYC3 protein sequence query.

Template ID	Total score	Sequence coverage	E-value	Identity (%)	Resolution (Å)
4YWC_A	498	MYC3: 5-242 JAZ9: 218-239	3e-176	100	2.10
5T0F_A	414	MYC3:44-242 JAZ10: 16-58	7e-144	100	2.15
4YZ6_A	407	MYC3:44-238 JAZ1: 200-221	6e-141	100	1.95
4RQW_A	398	MYC3:44-238	2e-137	98	2.2
5GNJ_G	122	MYC3:409-484	3e-33	78	2.7

**Figure 3:**
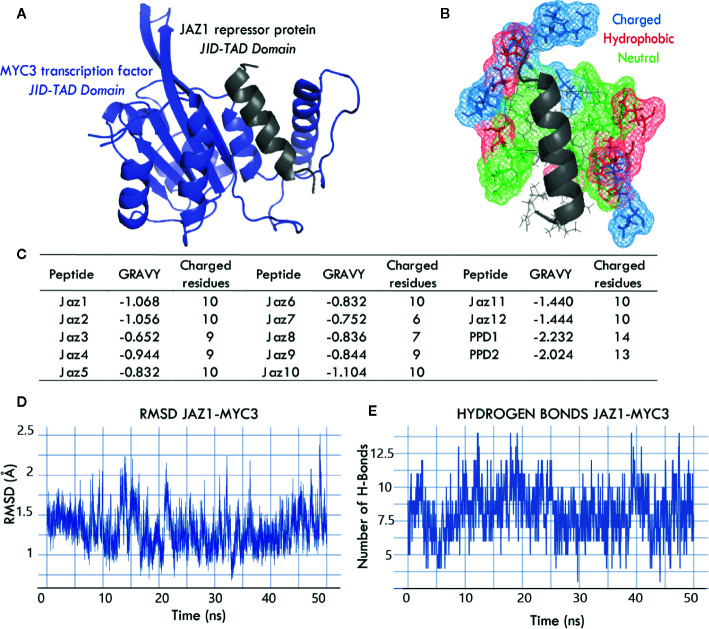
**(A, B)** Interaction domains between JAZ1 (Jas) and MYC3 (JID-TAD) proteins reveal a hydrophobic and charged pocket that surrounds the Jas degron; this coupling mechanism is similar in all 14 models. Charged, hydrophobic, and neutral amino acids are depicted in blue, red, and green, respectively. **(C)** The table shows the biochemical properties of Jas and Jas-like domains, GRAVY scores below 0 indicate that the binding interface in all JAZ/PPD-MYC3 complexes is highly hydrophobic. The number of charged residues within the domain indicates that the electrostatic interactions predominate in the interface. **(D)** The RMSD plot of JAZ1-MYC3 complex during the 50-ns molecular dynamics simulation indicates that the simulations reached the equilibrium. This behavior is observed for all 14 simulations. **(E)** Fluctuation of the number of hydrogen bonds of that JAZ1-MYC3 interface during the 50-ns molecular dynamics simulation shows that the H-bond network is stable, and that there is no uncoupling of the interface.

Using 4YWC_A and 4YZ6_A as templates, we assembled 14 3D models corresponding to the MYC3 JID-TAD domain interacting with Jas or Jas-liked domains of the 14 *Arabidopsis* *thaliana* class II TIFY proteins. **Figure 3A** shows one of the 14 models, i.e., the JAZ1-MYC3 complex. All models comprised the JID-TAD domain from the MYC3 transcription factor (**Figure 3A**, shown in blue) and Jas or Jas-like domain from the JAZ repression protein and PPD protein, respectively (shown in gray). **Figure 3B** shows the MYC3 binding pocket, which has predominantly charged and hydrophobic residues that surround the Jas helical domain of the JAZ protein. **Figure 3C** depicts in detail the number of charged residues and the GRAVY (Grand Average of Hydropathy) score of all 14 Jas and Jas-like domains (Doolittle, 1992; Young et al., 1994). In the GRAVY index, the larger the number, the more hydrophobic the average protein.

### JAZ-MYC3 and PPD-MYC3 Full-Length 3D Protein Structure Predictions Comply With Quality Metrics

The quantitative validation of the structural models can be found in **Table 2**. This validation was carried out using four servers that assess different biochemical and structural characteristics of the complexes. A three-step validation was implemented. The first validation (Raw Model) corresponds to the assessment of the initial model obtained immediately after the construction of the 3D structures by the online servers. The second validation was done for the energy-minimized structures, a MD process to stabilize the stereochemical characteristics of JAZ-MYC3 and PPD-MYC3 models. The third validation (Molecular Dynamics) was conducted on the models after the 50-ns molecular dynamics simulations, which were performed to create the appropriate physicochemical environment present in physiological conditions. **Table 2** reports the average quality scores and the corresponding standard deviation of the 14 3D models throughout the three-step validation.

**Table 2:** Protein structure validation of JAZ-MYC3 homology models.

Validation Step	Errat	ProQ		QMean4	SolvX	
		LGScore	MaxSub		JAZ	MYC3
**Raw Model**	85.15 +/-0.65	4.41 +/- 0.16	0.44 +/-0.013	0.83 +/- 0.003	-5.67 +/- 4.56	-34.48 +/- 0.86
**Energy Minimized**	95.89 +/-2.30	5.10 +/- 0.23	0.22 +/- 0.01	0.81 +/- 0.01	-5.68 +/- 3.55	-12.91 +/- 15.34
**Molecular Dynamics**	94.12 +/-2.81	4.68 +/- 0.35	0.17 +/-0.02	0.78 +/-0.01	-4.88 +/-3.96	-43.83 +/- 10.03

Errat quadratic error function evaluates nonbonded protein interactions using a quality factor, which ranges from 1%‑100%. Structures with a quality factor above 95% are considered good models (Colovos and Yeates, 1993). JAZ-MYC3 proteins after Molecular Dynamics simulation scored an average quality factor of 94.12 +/- 2.80%, which indicates high-quality models. This score was higher than the average scores obtained from the assessment of raw models and energy-minimized-only models (85.15+/-0.65 and 92.89+/-2.30, respectively). The ProQ quality evaluator uses neural networks to predict structural quality based on SASA, residue-residue, and atom-atom contacts (Wallner and Elofsson, 2003). It uses two scores, LGScore and MaxSub. The difference between both lies in the size of the protein complex to be evaluated. Larger complexes have a higher probability of getting high LGScores, while small complexes tend to score higher in the MaxSub metric. High-quality models have an LGScore above 1.5, and a MaxSub score above 0.1 (Wallner and Elofsson, 2003). JAZ-MYC3 complexes showed high quality in both metrics indistinctly from the validation step.

The QMean4 score determines the degree of “nativeness” of a protein model against experimental crystallographic structures. This quality evaluator does not depend on the size of the protein, unlike ProQ scoring functions. The QMean score ranges from 0 to 1. High-quality models usually score above 0.6 (Benkert et al., 2010). All JAZ-MYC3 models scored above 0.7, presenting small differences between validation steps. The SolvX analysis further supported the quality of our predicted 3D structures. This structural validation server determines proper folding through the calculation of the SASA as a degree of compactness (Holm and Sander, 1992). All predicted JAZ-MYC3 and PPD-MYC3 models present negative SolvX scores for every refinement step, which indicates proper folding of the tertiary structure throughout molecular dynamics processing.

Moreover, we evaluated the quality of molecular dynamics simulations. **Figures 3D, E** show molecular dynamics’ metrics, namely, RMSD (Root Mean Square Deviation) and hydrogen bonds profile along a 50-ns trajectory. RMSD remains stable throughout the simulation and fluctuates around 1‑2 Å. This behavior is expected in molecular dynamics simulations that reach stability. The hydrogen bonding profile varies around 6 to 10 hydrogen bonds between the Jas peptide and the MYC3 JID-TAD domain. The stability of the hydrogen bonding indicates a good coupling between structures, and this is a good predictor of binding affinity. Overall, JAZ-MYC3 and PPD-MYC3 models showed high-quality structural characteristics based on the assessment of four independent evaluators and stability throughout the 50-ns trajectory of MD simulations.

### JAZ-MYC3 Complexes Showed a Higher Binding Affinity Than PPD-MYC3 Complexes

There is not enough structural data that can guide us to a clear understanding of why PPD and JAZ proteins participate in widely different biological processes. The availability of 3D structures for these complexes is limited to JAZ1-MYC3, JAZ9-MYC3, and JAZ10-MYC3 complexes (Zhang et al., 2015). There are no crystal structures of PPD-MYC3 complexes. For this reason, we used an *in silico* approach to better understand the specificity of JAZ and PPD proteins during the recognition of their binding partner. We hypothesize that slight differences in binding affinity within the JAS domain versus the PPD’s Jas-like domain are due to the presence or absence of specific residues, hotspots, and may profoundly influence protein-protein recognition for the cases studied.

Provided the fact that computational methods allow us to study systems dynamically, we can calculate the binding affinity during each simulation step. By doing so, we could obtain a robust binding energy prediction comparable with experimental approaches. Therefore, we used Gromos algorithm with an RMSD cutoff of 0.25 nm to cluster the conformational space created during the 50-ns MD simulations of JAZ/PPD-MYC3 complexes (Berendsen et al., 1995). We used FoldX *AnalyseComplex* functionality to determine the free energy profile of each cluster representative (Schymkowitz et al., 2005). The cluster representatives were defined as the molecular conformations that appeared the most during MD simulation trajectories. Some cluster representatives were more frequent during MD than others. Therefore, to determine the binding energy of JAZ/PPD-MYC3 complexes, we calculated a weighted average of binding energy using the frequency of each cluster representative for each complex. **Table 3** shows the predicted van der Waals, electrostatic, polar, and apolar solvation energies which are the critical energy terms from which FoldX calculates the free binding energy (ΔG_binding_) for each complex (Schymkowitz et al., 2005). The 12 JAZ-MYC3 complexes showed an average ΔG_binding_ = -10.94 +/- 2,22 kcal/mol (negative value indicates favorable binding).

**Table 3:** Summary of the energy profile of JAZ-MYC3, PPD-MYC3, and mPPD-MYC3 complexes.

Complex	#Clusters	Binding Free Energy (kcal/mol)	Van der Waals (kcal/mol)	Electrostatic (kcal/mol)	Polar solvation (kcal/mol)	Apolar solvation (kcal/mol)
JAZ1-MYC3	9	-11.78 +/-0.021	-13.2 +/- 0.013	-4.22 +/- 0.001	15.57 +/- 0.029	-18.33 +/- 0.014
JAZ2-MYC3	11	-7.69 +/- 0.018	-13.29 +/- 0.008	-4.53 +/- 00.7	16.94 +/- 0.018	-17.67 +/- 0.008
JAZ3-MYC3	10	-10.46 +/-0.031	-14.09 +/- 0.009	-3.44 +/- 0.006	17.07 +/- 0.013	-18.61 +/- 0.013
JAZ4-MYC3	13	-13.31 +/- 0.044	-13.63 +/- 0.007	-4.23 +/- 0.006	16.85 +/- 0.013	-18.36 +/- 0.007
JAZ5-MYC3	11	-15.63 +/- 0.028	-15.34 +/-0.012	-5.88 +/- 0.011	19.1 +/- 0.023	-20.23 + /- 0.012
JAZ6-MYC3	14	-11.26 +/- 0.027	-14.74 +/- 0.007	-6.14 +/- 0.015	19.77 +/- 0.015	-18.69 +/- 0.011
JAZ7-MYC3	16	-10.75 +/- 0.021	-14.98 +/-0.041	-4.24 +/-0.013	19.39 +/- 0.083	-19.93 +/-0.041
JAZ8-MYC3	12	-10.67 +-/ 0.031	-15.31 +/-0.017	-4.25 +/- 0.012	20.1 +/- 0.028	-20.96 +/- 0.025
JAZ9-MYC3	14	-9.84 +/- 0.027	-14.9 +/- 0.02	-4.42 +/- 0.021	19 +/- 0.032	-19.7 +/- 0.022
JAZ10-MYC3	18	-12.35 +/- 0.025	-14.41 +/- 0.012	-4.05 +/- 0.009	18.35 +/- 0.026	-18.81 +/- 0.011
JAZ11-MYC3	20	-7.74 +/-0.043	-16.48 +/- 0.021	-4.35 +/- 0.014	21.88 +/-0.061	-19.84 +/- 0.02
JAZ12-MYC3	11	-9.75 +/- 0.079	-16.28 +/- 0.014	-5.48 +/- 0.012	22.64 +/- 0.041	-20.46 +/- 0.012
PPD1-MYC3	11	-5.32 +/-0.049	-13.82 +/- 0.019	-4.57 +/-0.015	19.74 +/- 0.025	-16.28 +/- 0.03
PPD2-MYC3	16	-7.35 +/-0.031	-15.09 +/- 0.007	-3.69 +/- 0.01	20.57 +/- 0.012	-19.13 +/- 0.008
mPPD1-MYC3	4	-16.42 +/- 0.006	-13.43 +/- 0.001	-3.17 +/- 0.002	16.28 +/- 0.001	-17.57 0.002
mPPD2-MYC3	4	-15.41 +/- 0.013	-13.34 +/- 0.002	-3.79 +/- 0.003	17.06 +/- 003	-17.2 +/- 0.001

In contrast, PPD1/2-MYC3 complexes showed a predicted average ΔG_binding_ of -6.34 +/- 1.44 kcal/mol (**Table 3**). Even though this value denotes favorable binding, ΔG_binding_ was significantly lower than the JAZ-MYC3 average ΔG_binding_. Remarkably, the PPD1-MYC3 complex presented one representative with a predicted positive binding energy of 1.05 kcal/mol (a positive ΔG_binding_ denotes unfavorable binding between two proteins). This representative accounts for 20.33% of the complete pool of structural binding modes for PPD1-MYC3 clusters. As shown, there are significant differences in binding energies between JAZ-MYC3 and PPD-MYC3 complexes. A way to find out the reason for these differences is to compare the per-residue energy contribution in JAZ-MYC3 and PPD-MYC3 interfaces.

Although PPD proteins display significant sequence similarity to JAZ proteins, they are not involved in JA signaling. JAZ and PPD proteins both contain a PEAPOD domain, a ZIM-domain, and a Jas/Jas-like domain in their protein structure (Bai et al., 2011). Alterations in the Jas-like domains of PPD1 (SL-YR-R motif) and PPD2 (SL-YL-R motif) are likely responsible for the nulling of the interaction between the PPD and MYC proteins. Thus, we decided to investigate further the contribution of each residue to the overall binding energy.

The Jas domain from JAZ proteins showed less exposed residues (6 to 10 residues) compared to Jas-like domains from PPD proteins (14 for PPD1 and 13 for PPD2). Jas binding domains present higher GRAVY scores compared with Jas-like domains, meaning that Jas domains are more hydrophobic than their counterparts (Jas-like domains).

### Hotspots Prediction Reveal a Short Linear Motif Which May Define JAZ-MYC3 Binding Specificity

With the cluster representatives structures (the more representative structures for each complex simulated, accounting 12 for each) obtained from the molecular dynamics trajectory, we performed computational scanning mutagenesis. We calculated the per-residue energy contribution (a weighted average of the cluster representatives), and with these results and available experimental data from the literature, we tested several machine learning classifiers. This approach allowed us to predict molecular hotspots based on both computational calculations and experimental data, which reinforce the accuracy of the predictions.

We used a supervised learning approach to build classifiers using several machine learning algorithms. The training data set comprises deleterious and non-deleterious mutations collected from the literature (Melotto et al., 2008; Withers et al., 2012; Zhang et al., 2015). We considered only single point mutations to generate the training set. We calculated the change in binding energy (ΔΔG _binding_) using computational scanning mutagenesis for all the 20 natural amino acids (**Table S1**). Using this data set, we trained, cross-validated, and tested several machine learning classifiers. The performance of the best classifiers is shown in **Table 4**.

**Table 4:** Quantitative assessment of the best ranked machine learning algorithms for JAZ-MYC3 hot spots prediction.

Algorithm	TP Rate	FP Rate	Precision	Recall	F-Measure	MCC	ROC Area	PRC Area	Class
**Random Forest**	0.818	0.125	0.900	0.818	0.857	0.685	0.943	0.959	NDM
	0.875	0.182	0.778	0.875	0.824	0.685	0.943	0.943	DM
	0.842	0.149	0.849	0.842	0.843	0.685	0.943	0.952	Average
**Sequential Minimal Optimization**	1.000	0.250	0.846	1.000	0.917	0.797	0.875	0.846	NDM
	0.750	0.000	1.000	0.750	0.857	0.797	0.875	0.855	DM
	0.895	0.145	0.911	0.895	0.892	0.797	0.875	0.850	Average
**Multilayer Perceptron**	0.818	0.125	0.900	0.818	0.857	0.685	0.920	0.940	NDM
	0.875	0.182	0.778	0.875	0.824	0.685	0.920	0.924	DM
	0.842	0.149	0.849	0.842	0.843	0.685	0.920	0.934	Average
**Naïve Bayes**	0.909	0.125	0.909	0.909	0.909	0.784	0.943	0.954	NDM
	0.875	0.091	0.875	0.875	0.875	0.784	0.943	0.952	DM
	0.895	0.111	0.895	0.895	0.895	0.784	0.943	0.953	Average

TP Rate, True positive rate; FP Rate, false positive rate; MCC, Matthews correlation coefficient; ROC Area, Receiver Operating Characteristic area under the curve; PRC Area, Precision Recall area under the curve. Each score ranges from 0 to 1, 1 being the best possible outcome. The training data set was divided into two classes NDM (non-deleterious mutations) and DM (deleterious mutations).

We used several quality metrics to evaluate the performance of the classifiers. For instance, we checked the true-positive rate, false-positive rate, precision, recall, F-Measure, Matthew correlation coefficient, ROC area, and PRC area for each classifier. For most metrics, a value close to one is characteristic of a good model, except for the false-positive rate where values close to zero are the best. The Matthew correlation coefficient index goes from -1, indicating absolutely no correspondence with the data, over zero, meaning no better than pure chance, to +1, indicating a perfect correspondence between the model and the data. The top-scoring models, according to these quality metrics, were random forest, sequential minimal optimization, multilayer perceptron, and naïve Bayes (**Table 4**). The most common descriptors used by the classifiers were alanine's, isoleucine's, lysine's, proline's, serine's, and valine’s ΔΔG_binding_ energy (**Table S1**). It was clear that alanine would be one of the most informative features because alanine scanning mutagenesis is the gold standard for hotspot discovery.

**Figure 4:**
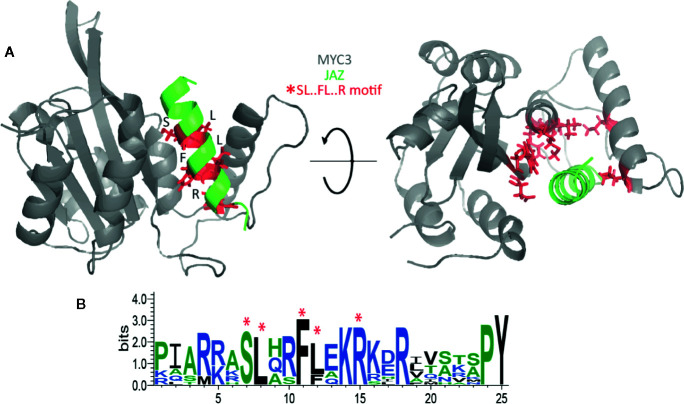
**(A)** Spatial location of the SL.FL.R motif in the JAZ-MYC3 complex. Red sticks represent the small binding motif. **(B)** Multiple sequence alignment logos representation of the Jas domain. Red asterisks denote the position of the SL.FL.R motif.

We chose the sequential minimal optimization (SMO) classifier for assessing JAZ-MYC3 and PPD-MYC3 models because it outperforms other classifiers with 89.5% instances correctly classified as a given class. Also, this classifier model was more conservative than the other classifier tested because the SMO was most likely to classify a residue as non-deleterious (not a hotspot) than as deleterious. To be a conservative model is a desirable behavior since only a few residues in a protein will be hotspot. The PRC area average for the classifier was still very high (0.856) overall for a conservative model classifier. The analysis of the classifier predictions highlighted the importance of a conserved hotspot motif within JAZ-MYC3 complexes. This motif was formed of five specific residues within the linear motif Serine-Leucine-X-X-Phenylalanine-Leucine-X-X-Arginine amino acids (SL.FL.R), X being any residue. SL.FL.R, which are in direct contact with the binding pocket of the MYC3 through hydrogen bonds and van der Waals interactions (**Figure 4A**). Therefore, this hotspot motif highly contributes to the binding affinity between JAZ proteins and MYC3 transcription factor. **Figure 4B** shows a logo of Jas domain residues. Highly conserved amino acids are represented by bigger letters. The SL.FL.R motif is a highly conserved motif within JAZ-MYC3 complexes. Notably, not all conserved residues are hotspots for specific protein-protein interfaces; however, hotspots are usually conserved residues.

### PPD-MYC3 Models Differ at Their Protein Interface

**Figure 5:**
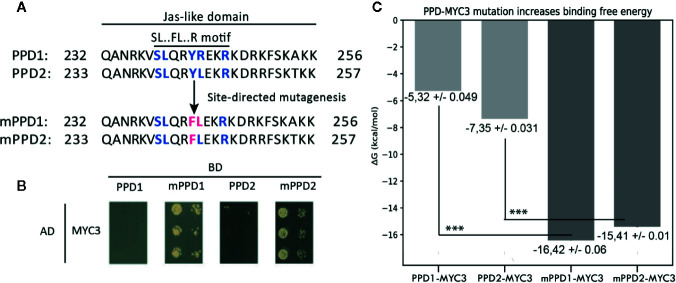
Site-directed mutagenesis assay of PPD-MYC3 complexes. **(A)** Amino acid alignment showing chances in the Jas-like domain of PPD1, PPD2, and mutants mPPD1 and mPPD2. **(B)** Yeast-two hybrid experiments are showing the favorable change in affinity upon mutation of the SL.FL.R motif in PPD1 and PPD2. **(C)** Predicted free binding energy of PPD1, PPD2, and mPPD1, mPPD2 mutants. Triple asterisks show statistical significance (p < 0.05) between wild type and mutants.

PPD1&2 proteins are not reported to interact with MYC3, even though they have a Jas-like domain similar to JAZ proteins. When looking at the PPD Jas-like domain, the linear motif identified for the Jas-domain is incomplete, which may be the reason why PPD proteins do not interact with MYC3. To confirm this hypothesis, we performed a yeast two-hybrid (Y2H) assay using “wild-type” PPDs and mutant PPDs (mPPDs) with a mutation in the Jas-like domain (mPPD1 and mPPD2; **Figure 5A**) to investigate the effect of these mutations for the interaction between PPDs and MYC3. **Figure 5B** depicts the Y2H assay. As reported before, and as supported by the binding energy predictions described above, PPD1 and PPD2 do not interact with MYC3. However, both mPPD1 and mPPD2 present binding affinity towards MYC3 (**Figure 5B**), establishing the fundamental role of the identified linear motif in the interaction.

In parallel, we compared if the Y2H results could be corroborated using computational methods that calculate the predicted free binding energy between MYC3 and mPPD1/mPPD2. Indeed, we found a low predicted free binding energy between MYC3 and the wild-type PPDs, pointing to a weak or no interaction capacity between these proteins. Conversely, we found a significantly higher free binding energy, thus suggesting a higher interaction affinity between MYC3 and the mPPDs mutants. The artificially reestablished SL.FL.R linear motif provokes a three-fold increase in binding energy (ΔG_binding_) for PPD1 (from -5.32 +/- 0.049 kcal/mol to -16.42 +/- 0.031 kcal/mol) and a two-fold increase for PPD2 (from -7.35 +/- 0.031 kcal/mol to -15.41 +/- 0.013 kcal/mol) (**Figure 5C**). The difference in binding energy is statistically significant (p<0.05).

Overall, molecular hotspot prediction and computational analysis explained why the SL.FL.R linear motif is responsible for most of the binding dynamics. This conserved linear motif may indeed be the molecular fingerprint that MYC3 uses to recognize and bind to JAZ proteins instead of PPD proteins. Site-directed mutagenesis and Y2H assays confirm the importance of the SL.FL.R linear motif in for MYC3 protein recognition, and it is surprising that by inducing specific punctual mutations in the PPD wild-type domain, a novel interaction with MYC3 can be induced for PPD proteins. This simple change had a real impact on the binding energetics of PPD proteins, which was also found using computational methods.

## Discussion

The study of protein families as a whole system is one of the priorities of modern protein research. Particularly in plants, it is relatively common to find extended protein families throughout their genome, and a problem arises in the fact that for most of these families, their functions or structure are poorly characterized. Several experimental and computational approaches have played a significant role in addressing this question. Experimental techniques like site-directed mutagenesis are widely used for describing binding interfaces at a molecular level.

Nevertheless, the costs and effort associated with these experiments are high, and experimental techniques are limited to small-scale tests (Zinzalla and Thurston, 2009). Several computational approaches, on the other hand, such as molecular dynamics and machine learning predictors, usually required high amounts of computational resources and time to get accurate results. Moreover, the correlation between experimental and computational predictions has generally been low because of experimental and computational shortcomings, but it has been improving over time (Cukuroglu et al., 2014). There are no general criteria to experimentally define what a hotspot is. The same can be a problem for the computational prediction that relies on a specific threshold change in free binding energy (Liu et al., 2018). From the computational point of view, there are only small sets of experimental data available to train the models and too many potential features so that overfitting is possible when using machine learning approaches (Liu et al., 2018).

The advantage of using our methodology is that we used available experimental data as a scaffold to design computational experiments that increase the accuracy of our predictions. We collected data for a specific protein-protein interaction from the literature and predicted the effect on the whole group of proteins for which minimal experimental data was available. Integrating computational insights to characterize or design complex system increases our understanding and optimizes experimental resources (Montero-Oleas et al., 2018; Ryan et al., 2019). By doing so, we can escalate the study of protein-protein interactions to specific families of proteins, as we did with the *Arabidopsis* TIFY class II family. We suggest that our *in silico* strategy is suitable for the study of plant family proteins because gene duplication is very extended in plant lineage members, for example, on average 65% of annotated genes from a group of 41 sequenced plant genomes had duplicated copies (Panchy et al., 2016). Redundant protein families include, but are not limited to, transcription factors, membrane proteins, peptidases, Cytochrome P450, or signaling proteins (Roskoski, 2012; Bolger et al., 2018; Seternes et al., 2018).

This gene duplication process can allow the acquisition of novel functions, interactions, or expression patterns that could confer new characteristics for the benefit of living organisms (Rensing, 2014). Usually, these evolutionary novelties are related to new molecular functions, plant structures, and adaptive traits (Hanada et al., 2008). By studying protein families with our methodology, we can identify and determine the molecular reasons why the small molecular differences make possible the diversity of functions and interactions seen within a family of interest.

Automated full-length 3D protein structural predictions have been shown to be very accurate, especially when there are similar proteins available in the structural databases used by the software (Aguilera-Pesantes and Méndez, 2017). Since the partial structures of JAZ1, JAZ9, and JAZ10 of *Arabidopsis* *thaliana*, interacting with MYC3, are available, this allowed the server to generate suitable starting full-length 3D structures. In the second stage, we calculated the free binding energy of each of the complexes to evaluate the importance of each residue at the protein-protein interface. These calculations allowed us to rank the contribution of each residue to the binding since not all residues contribute equally to the interaction between two proteins. Those residues that contribute more are considered hotspots residues, and their biological relevance needs to be highlighted since a mutation in these residues will, in general, significantly decrease and even abolish the interaction between two proteins.

In the third stage, using the information calculated *in silico* and the known experimental data (site-directed mutagenesis data), we were able to train machine learning algorithms. The aim was to recognize from all residues in the protein, which ones were hotspots for the specific protein-protein interface. This information is valuable to explain why we observe a weakening of the protein-protein interaction in some instances. Specifically, a weaker interaction is present in the *in silico* experiments as well as in the *in vitro* experiments for wild-type PPD proteins that lack the complete linear motif we derived from the consensus sequence identified from the machine learning classifier results. Therefore, identifying the consensus of these hotspots along all the TIFY class II family proteins allowed us to suggest a linear binding motif for the all JAZ-MYC3 complexes. Finally, our explanation of the Y2H results is that slight differences in the binding affinity due to specific residues (hotspots identified by the consensus from our machine learning classifier results) may profoundly impact protein-protein recognition and interface stabilization.

The approach is not designed to be used as a protein-protein interaction discovery tool directly. But, its significance could be that if one protein-protein interaction is discovered, the interaction with all the other proteins of the same family can be rapidly tested *in silico.* With this in mind, it could also be used to test a hypothesis of a novel protein-protein interaction. First, a known protein-protein interaction must exist, and from there, similar proteins can be searched with other methods (Zhang et al., 2010). To get a stronger sense to decide if these new proteins could interact, the researcher could use the presented approach to look for hotspots. If there are several hotspots present in the tested interface, the data may help to validate the original hypothesis and lead to an experimental test to verify the protein-protein interaction. Regarding the protein similarity needed to apply this method, it has been observed that there is a conservation of interface locations at the family and superfamily levels and that even there is some conservation with remote structural neighbors (Korkin et al., 2005; Littler et al., 2005; Zhang et al., 2010). Also, there is structure conservation of protein structure till the “twilight zone” around 20%‑35% homology, and even at lower percentage homology for transmembrane proteins (Olivella et al., 2013). These considerations suggest that there will be many protein families for which protein-protein interfaces could be studied with ours or similar approaches.

The assessment of the *Arabidopsis* *thaliana* TIFY class II group is an excellent example of how small molecular changes can lead to significant differences in the interaction between proteins. JAZ proteins interact with the MYC3 transcription factor through a short helical domain (Jas domain). On the other hand, PPD proteins exhibit a Jas-like domain pretty similar to JAZ proteins, but they do not interact with MYC3 (Gonzalez et al., 2015). The question that arises is how the MYC3 transcription factor specificity works despite the striking similarities between JAZ and PPD proteins. Using a computational approach which included modeling, molecular dynamics, and a machine learning classification models, we discovered a short linear binding motif that may be the clue of JAZ-MYC3 binding specificity.

The SL.FL.R linear motif, which is absent in PPD proteins, interacts with the binding pocket of the MYC3 transcription factor, which is formed by hydrophobic and charged amino acids. This linear motif is composed of five molecular hotspots, meaning that binding energy of the complex upon mutation of one of them will considerably decrease compared to the mutation of other residues. As a proof of concept, we developed an experiment that consisted of completing the SL.FL.R linear motif at the Jas-like domain of wild-type PPD proteins resulting in that this change made PPD proteins able to bind MYC3. Surprisingly, mutant PPD1 and PPD2 formed a complex with the MYC3 transcription factor with a binding energy that falls in the same range of JAZ-MYC3 complexes.

The coupling of computational predictions using relevant and specific experimental data will result in a higher accuracy of hotspot predictions compared with generic computational protein-protein prediction assays, and the method could generate results compared more quickly to experimental techniques. Besides, we suggest that this methodology can be translated to other protein families with unknown interacting partners and which share at least 30% of homology in their sequence, or even at lower percentage homology for membrane proteins (Sander and Schneider, 1991; Olivella et al., 2013). This homology requisite is necessary for developing an accurate full-length 3D structural prediction. Besides, it is an asset to have a crystal structure of one of the protein family members; however, it is not crucial. Moreover, in the case of the JAZ-MYC3 protein complex, we know their binding conformation. Nevertheless, in the absence of that information, we could have predicted it using protein-protein docking software. Overall, our methodology allows us to predict molecular hotspots and their contribution to the global binding energy quickly and robustly.

## Data Availability Statement

The raw data supporting the conclusions of this article will be made available by the authors, without undue reservation, to any qualified researcher.

## Author Contributions

MA helped with the planning of the computational experiments, participated in the writing and editing of the manuscript. AL-R and AG participated in the analysis and interpretation of results, as well as writing and editing the manuscript. SO and SA-R performed the computational experiments, analyzed data, and helped with preparing graphics. SO also participated in the writing and editing of the manuscript. JG and LP performed the wet-lab experiments, analyzed the corresponding data, made the images, and wrote the wet lab experimental’s methods and the results sections.

## Funding

This research was supported by funding from the Agency for Innovation by Science and Technology in Flanders (IWT) for a predoctoral fellowship to JG and the Research Foundation Flanders through the project G005312N and a postdoctoral fellowship to LP. The computational research was funded and supported through the USFQ Chancellor grant granted to MA.

## Conflict of Interest

The authors declare that the research was conducted in the absence of any commercial or financial relationships that could be construed as a potential conflict of interest.
