# Supplementary figures and images for: The Molecular Basis of JAZ-MYC Coupling, a Protein-Protein Interface Essential for Plant Response to Stressors
Source: Front Plant Sci. 2020 Aug 20;11:1139. doi: 10.3389/fpls.2020.01139 (PMC7468482; doi:10.3389/fpls.2020.01139)

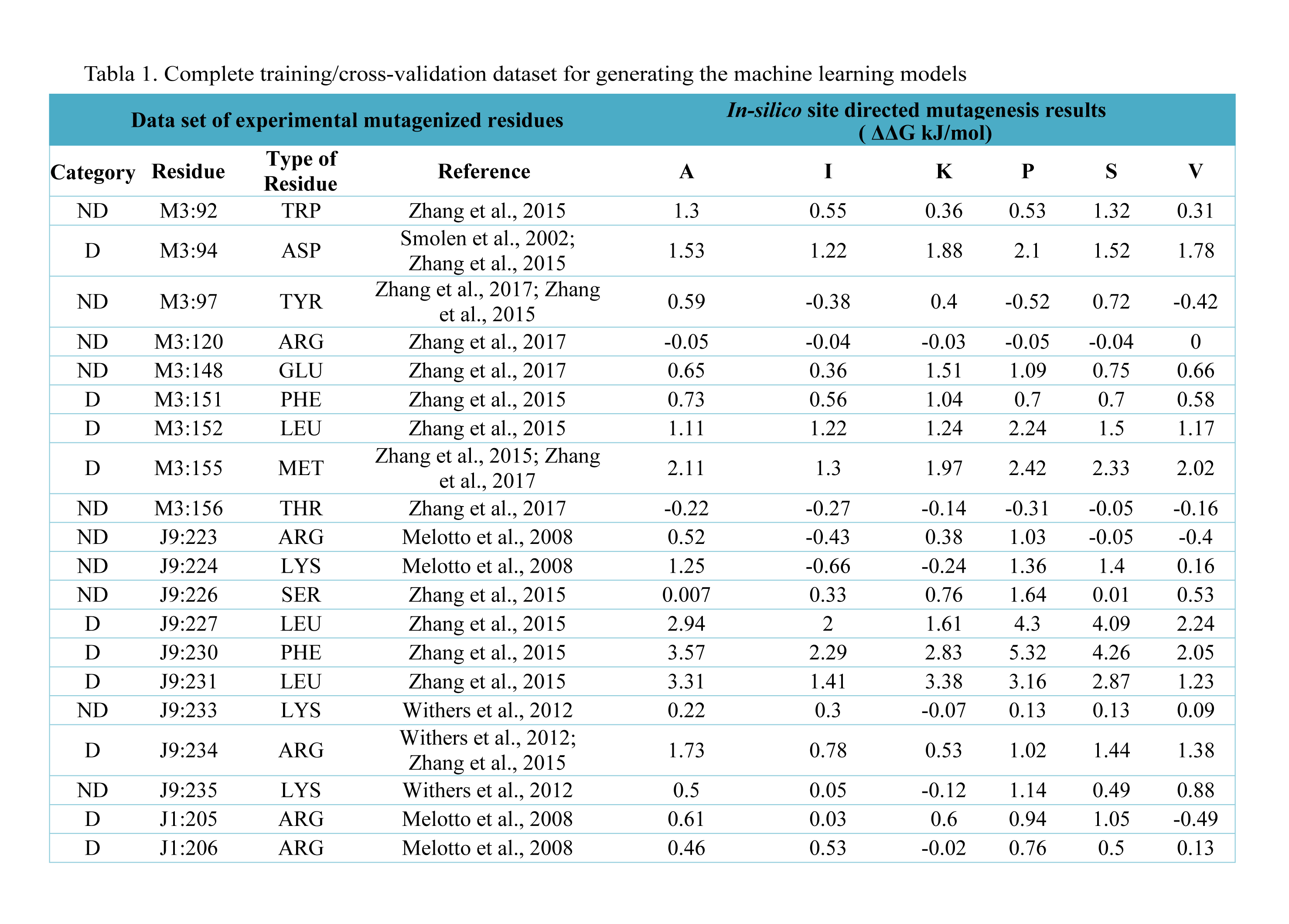

Supplement: Supplementary file 1 [file Image_1.tif]
